# Structural basis for light control of cell development revealed by crystal structures of a myxobacterial phytochrome

**DOI:** 10.1107/S2052252518010631

**Published:** 2018-08-29

**Authors:** Nicole C. Woitowich, Andrei S. Halavaty, Patricia Waltz, Christopher Kupitz, Joseph Valera, Gregory Tracy, Kevin D. Gallagher, Elin Claesson, Takanori Nakane, Suraj Pandey, Garrett Nelson, Rie Tanaka, Eriko Nango, Eiichi Mizohata, Shigeki Owada, Kensure Tono, Yasumasa Joti, Angela C. Nugent, Hardik Patel, Ayesha Mapara, James Hopkins, Phu Duong, Dorina Bizhga, Svetlana E. Kovaleva, Rachael St. Peter, Cynthia N. Hernandez, Wesley B. Ozarowski, Shatabdi Roy-Chowdhuri, Jay-How Yang, Petra Edlund, Heikki Takala, Janne Ihalainen, Jennifer Brayshaw, Tyler Norwood, Ishwor Poudyal, Petra Fromme, John C. H. Spence, Keith Moffat, Sebastian Westenhoff, Marius Schmidt, Emina A. Stojković

**Affiliations:** a Department of Biology, Northeastern Illinois University, Chicago, IL, USA; b Department of Biochemistry and Molecular Genetics, Feinberg School of Medicine, Northwestern University, Chicago, IL, USA; c Department of Physics, University of Wisconsin, Milwaukee, WI, USA; d Department of Chemistry and Molecular Biology, University of Gothenburg, 40530 Gothenburg, Sweden; e Department of Biological Sciences, Graduate School of Science, University of Tokyo, 2-11-16 Yayoi, Bunkyo-ku, Tokyo, 113-0032, Japan; f Center for Applied Structural Discovery, Arizona State University, 85287 Tempe, AZ, USA; g RIKEN SPring-8 Center, 1-1-1 Kouto, Sayo-cho, Sayo-gun, 679-5148 Hyogo, Japan; h Department of Cell Biology, Graduate School of Medicine, Kyoto University, Yoshidakonoe-cho, Sakyo-ku, Kyoto 606-8501, Japan; i Department of Applied Chemistry, Graduate School of Engineering, Osaka University, 2-1 Yamadaoka, Suita, Osaka 565-0871, Japan; j Japan Synchrotron Radiation Research Institute, 1-1-1 Kouto, Sayo-cho, Sayo-gun, Hyogo 679-5198, Japan; k Faculty of Medicine, Anatomy, University of Helsinki, 00014 Helsinki, Finland; l Nanoscience Center, Department of Biological and Environmental Sciences, University of Jyvaskyla, 40014 Jyvaskyla, Finland; m Department of Biochemistry and Molecular Biology, The University of Chicago, Chicago, IL, USA

**Keywords:** phytochromes, photoreceptors, photosynthetic bacteria, myxobacteria, absorption spectra

## Abstract

Phytochromes are red-light photoreceptors that were first characterized in plants, with homologs in photosynthetic and non-photosynthetic bacteria known as bacteriophytochromes (BphPs). Upon absorption of light, BphPs interconvert between two states denoted Pr and Pfr with distinct absorption spectra in the red and far-red. They have recently been engineered as enzymatic photoswitches for fluorescent-marker applications in non-invasive tissue imaging of mammals. This article presents cryo- and room-temperature crystal structures of the unusual phytochrome from the non-photosynthetic myxo­bacterium *Stigmatella aurantiaca* (SaBphP1) and reveals its role in the fruiting-body formation of this photomorphogenic bacterium. SaBphP1 lacks a conserved histidine (His) in the chromophore-binding domain that stabilizes the Pr state in the classical BphPs. Instead it contains a threonine (Thr), a feature that is restricted to several myxobacterial phytochromes and is not evolutionarily understood. SaBphP1 structures of the chromophore binding domain (CBD) and the complete photosensory core module (PCM) in wild-type and Thr-to-His mutant forms reveal details of the molecular mechanism of the Pr/Pfr transition associated with the physiological response of this myxobacterium to red light. Specifically, key structural differences in the CBD and PCM between the wild-type and the Thr-to-His mutant involve essential chromophore contacts with proximal amino acids, and point to how the photosignal is transduced through the rest of the protein, impacting the essential enzymatic activity in the photomorphogenic response of this myxobacterium.

## Abstract 2

Light control of cell development is revealed by phytochrome structures of Myxobacteria.

## 1. Introduction   

**Figure 1:**
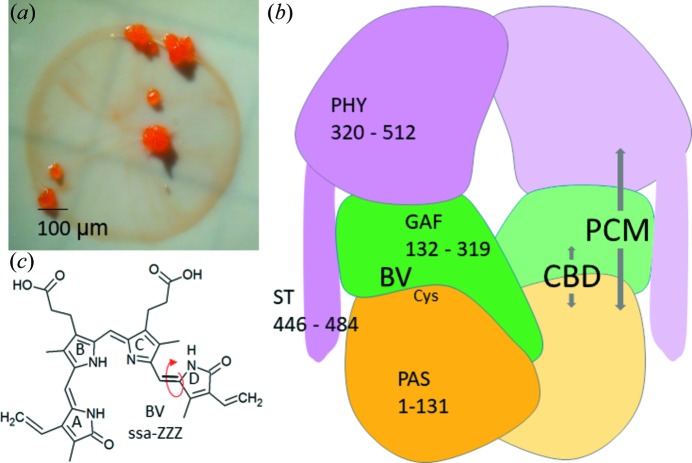
Myxobacterial fruiting bodies, and the architecture of BphPs. (*a*) *S. aurantiaca* fruiting bodies formed on filter paper in starvation media. (*b*) The architecture of BphPs. The PCM consists of PAS, GAF and PHY, and the CBD consists of PAS and GAF domains. The chromophore binds near the PAS and GAF domains, and is bound to a Cys. ST is the sensory tongue which probes the configuration of the BV chromophore and transmits the signal to the PHY domain. Amino-acid sequence numbers are given for the SaBphP1 (Huntley *et al.*, 2011 ▸). (*c*) The BV chemical structure found in the *syn*-*syn*-*anti* *ZZZ* configuration in the dark-adapted Pr state. The *Z* to *E* isomerization takes place at the double bond marked by the red arrow.

Non-photosynthetic myxobacteria are distinguished among prokaryotes by a multicellular stage in their life cycle known as fruiting bodies (Fig. 1 ▸ *a*), which in *Stigmatella aurantiaca* and the related *Chondromyces apiculatus* are stimulated by light (Qualls *et al.*, 1978 ▸; Müller *et al.*, 2006 ▸), a phenomenon that was demonstrated in the late 1970s (White *et al.*, 1980 ▸; Bhoo *et al.*, 2001 ▸; Davis *et al.*, 1999 ▸). Myxococcal fruiting bodies differ in complexity and range from 50 to 500 µm in size (Fig. 1 ▸ *a*), large enough to be viewed by compound light microscopy (Huntley *et al.*, 2011 ▸; Qualls *et al.*, 1978 ▸). *S. aurantiaca* contains two previously uncharacterized bacteriophytochromes (BphPs) denoted SaBphP1 and SaBphP2, which may be responsible for the light-stimulated growth of multicellular fruiting bodies. Phytochromes are red-light photoreceptors found in various plants and microorganisms. Although their role is critical and well understood in plants and photosynthetic bacteria, their physiological function in non-photosynthetic bacteria remains largely unknown (Fixen *et al.*, 2014 ▸; Giraud *et al.*, 2002 ▸, 2005 ▸). BphPs consist of three domains denoted PAS, GAF and PHY (see Fig. 1 ▸ *b*), and an effector domain which is covalently attached to the PHY domain (Auldridge & Forest, 2011 ▸). Typically, the GAF domain harbors a covalently bound bilin chromophore, biliverdin (BV) (Figs. 1 ▸ *b* and 1*c*) and a heme-derived, open-chain tetrapyrrole (pyrrole rings *A*–*D*). Phycocyanobilin, PCB, is found in cyanobacterial phytochrome (Cph1) and phytochromobilin (PΦB) in plant phytochromes. Absorption of light by the BV chromophore causes its isomerization (Fig. 1 ▸ *c*) that generates extensive structural changes, *i.e.* a signal, which are transmitted to an output or effector module such as the spatially distant histidine kinase (HK) domain. One unusual conserved structural motif of unknown function is a knot at the interface of the PAS and GAF domains, where ∼35 N-terminal PAS domain residues thread through a loop made by amino acids of the GAF domain (Wagner *et al.*, 2005 ▸). The classical BphPs switch between a red absorbing state denoted Pr (λ_max_ ≃ 700 nm) and a far-red absorbing state denoted Pfr (λ_max_ ≃ 750 nm). Although absorption maxima may slightly vary between the BphPs (Auldridge & Forest, 2011 ▸), differences are typically 50–70 nm. Rotation about single bonds brings the individual pyrrole rings closer in the so-called ‘*syn*’ configuration or further apart, in the ‘*anti*’ configuration, which results in either a bent (*syn*) or a more linear (*anti*) geometry (Fig. 1 ▸ *c*). Absorption of a photon results in *Z* to *E* isomerization about the C15=C16 double bond between bilin rings *C* and *D*, and a clockwise rotation of ring *D* (Rockwell *et al.*, 2009 ▸). The photoswitch’s conformational changes are controlled by a hydrogen-bond network between conserved amino acids present in the GAF domain, ring *D* and propionate side chains of BV. The molecular basis of the Pr to Pfr photoconversion and the nature of the hydrogen-bond network that stabilizes ring *D* in the respective *Z* or *E* configuration, is not well understood at the atomic level.

The SaBphP1 and other myxobacterial BphPs lack a conserved histidine (His) at position 289 in the GAF domain (Mathes *et al.*, 2015 ▸). In the classical BphPs, the equivalent His forms a critical hydrogen bond to the BV ring *D* that stabilizes the Pr state. In bathy BphPs such as the PaBphP from *Pseudo-monas aeruginosa,* whose thermally stable dark-adapted state is Pfr (Yang *et al.*, 2008 ▸), the equivalent His forms a hydrogen bond to the *C*-ring propionate of the BV chromophore. If a variant is introduced at this position, classical and bathy BphPs show impaired photoactivity or stability (Mathes *et al.*, 2015 ▸; Wagner *et al.*, 2008 ▸; Yang *et al.*, 2008 ▸), thus emphasizing the importance of the conserved His in stabilizing Pr and Pfr states. SaBphP1 contains a threonine (Thr289) at this position, which is replaced by glycine in some myxobacterial BphPs (Appendix *A*).

In order to understand the role of the unusual Thr289 in signal transduction in SaBphP1, we replaced it with a His so that the T289H variant SaBphP1 resembles classical BphPs. We determined crystal structures of the SaBphP1 chromophore binding domain (containing PAS and GAF domains, Fig. 1 ▸ *c*) and the larger photosensory core module (containing the PAS-GAF-PHY domains, Fig. 1 ▸ *c*) in their wild-type and T289H forms. The four structures revealed details of how the addition of the PHY domain and/or the T289H variant stabilizes the quaternary structure of the SaBphP1 dimer, and the overall hydrogen-bonding network of conserved residues and BV in the chromophore binding pocket of the GAF domain. Furthermore, we place our results in the context of physiological responses of light-dependent starvation experiments in *S. aurantiaca* observed nearly 40 years ago, and predict details of the mechanism of signal transduction from the chromophore pocket into the PHY domain following isomerization of BV. We use the notation of *Z* or *E* for distinguishable BV configurations which are characterized by the *cis* or *trans* isoforms of the C15=C16 double bond at ring *D*; and use the terms Pr and Pfr for protein conformations which are caused by the *Z* to *E* isomerization and described directly by recently published X-ray structures (Burgie, Bussell *et al.*, 2014 ▸; Takala *et al.*, 2014 ▸; Burgie *et al.*, 2016 ▸). Thus, we distinguish between the *E* or *Z* isoforms of the chromophore ring *D*, and protein structures of long-lived (stable) Pr and Pfr states. This distinction becomes essential when interpreting UV–vis absorption spectra of SaBphP1 wild-type and mutant variants. We have identified amino acids important for the BphP photochemistry using absorption spectra of specific mutants in the GAF and PHY domains, and comparing the SaBphP1 Pr structures to published BphP structures determined in the Pr and/or Pfr states. In order to assess the impact of both temperature and X-ray dose on the four cryo structures of SaBphP1, we also report the ambient-temperature structure of the SaBphP1 PCM (T289H) mutant, essentially free of radiation damage (Lomb *et al.*, 2011 ▸) as a result of using an X-ray free electron laser (XFEL).

## 2. Methods   

Fruiting-body growth of *S. aurantiaca* was stimulated under starvation conditions by illumination with light of blue, red, and far-red wavelengths. Various SaBphP1 CBD and PCM constructs were cloned, overexpressed and purified for spectro­scopic and crystallographic investigations. Of these, the wild-type and the T289H mutant forms were crystallized. X-ray data were collected at cryo temperatures at beamline 19-ID-D of the Advanced Photon Source (APS), Argonne, USA. The PCM T289H room-temperature data were collected at beamline BL3 of SACLA, RIKEN SPring-8 Center, Japan. Structures were solved by molecular replacement. Pr and Pfr absorption spectra of constructs were determined after illumination by light of 660 or 700 nm (Pr) and 740 nm (Pfr) as appropriate.

### 2.1. Fruiting-body formation of *S. aurantiaca*   

**Figure 2:**
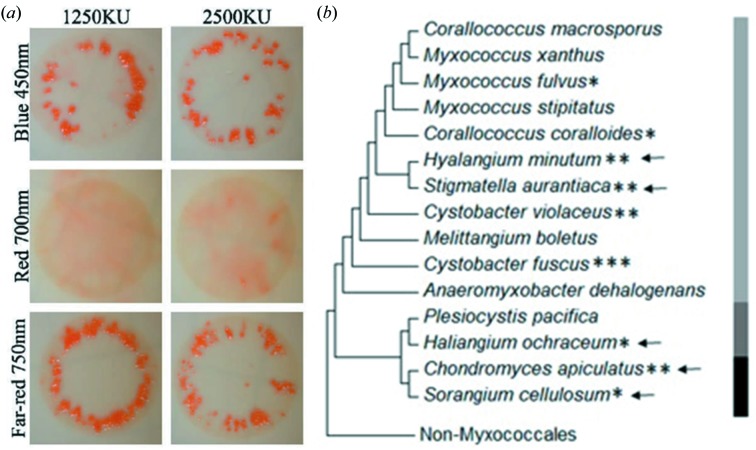
Fruiting-body formation in *S. aurantiaca*, and phylogenetic tree of Myxococcales. (*a*) Comparison of fruiting-body formation with (from top to bottom) blue (450 nm), red (700 nm) and far-red (750 nm) light illumination. Cell concentration is expressed in Klett Units (KU). (*b*) Phylogenetic comparison of Myxococcales indicating strains coding for BphPs with an asterisk (the number of asterisks indicates the number of BphPs) and photoactive yellow protein (PYP) with arrows. The side bar highlights three different suborders of Myxococcales: Cystobacterineae (light gray), Nanocystineae (dark gray) and Sorangineae (black). BphPs and PYPs are found in select species within all three suborders of Myxobacteria; the number and type of photoreceptors present varies between species.

Tryptone liquid media was inoculated with *S. aurantica* cells grown on tryptone agar media for 2–4 days at 305 K and grown to an OD at 660 nm of ∼0.5. Cells were washed in 0.1 *M* HEPES buffer pH 7.2 two times, concentrated to 1250 and 2500 Klett units (KU), spotted in 10 µl volumes on filter paper adhered to Wasseragar (Silakowski *et al.*, 1998 ▸) and dried before placing in a 305 K incubator under continuous illumination with either 450, 700 or 750 nm LED light, or in complete darkness. Cells were imaged using a Zeiss Stemi 508 compound light microscope equipped with a Zeiss Axiocam Erc 5s camera at 30× magnification. Growth of fruiting bodies (Fig. 2 ▸ *a*) were checked on the fifth and ninth day of incubation.

### 2.2. Phylogenetic analyses of Myxococcales   

The phylogenetic analysis was completed on 15 Myxococcales species that have had their complete genome sequenced. Representatives from other purple bacteria subgroups (non-Myxococcales) were used to show the root of the Myxococcales tree. 16S rRNA sequences were collected from the GenBank and placed into the *ClustalW*2 program (Thompson *et al.*, 2002 ▸) for multiple sequence alignment. Poorly aligned sequences at the ends were removed to fully extend both ends evenly and sequences were realigned. The alignment was used to create a maximum-likelihood tree with the *MEGA*6 program (Tamura *et al.*, 2013 ▸).

### 2.3. Cloning, overexpression and purification of the SaBphP1-CBD, PAS-GAF, and the SaBphP1-PCM, PAS-GAF-PHY   

The coding region for residues 1–328 (SaBphP1-CBD) and residues 1–516 (SaBphP1-PCM) of the wild-type SaBphP1 were PCR-amplified from *S. aurantiaca* DW4/3–1 genomic DNA and cut by restriction enzymes NdeI and HindIII (New England Biolabs, Beverly, USA), and ligated into the corresponding sites of the expression vector pET28*c*(+) (Invitrogen, Carlsbad, CA). The following primers 5′-CACCAGCATATGAGCACTGAGGCGTCCCGGAGC-3′ (forward) and 5′-CCAAAGCTTAGCGCTGGTCATAGT­CCTCGT-3′ (reverse), and 5′-CCAAAGCTTAGAGCAGTT­CCTCGCTGCGCT-3′ (reverse) were used to PCR-amplify the coding region of SaBphP1-CBD and SaBphP1-PCM, respectively. Site-directed mutagenesis to prepare the Thr289His (T289H) mutant and all other mutants described in this manuscript was carried out using the QuikChange Site-Directed Mutagenesis Kit (Agilent Technologies, Santa Clara, USA). The constructed plasmids and the pET11*a* vector carrying heme oxygenase were transformed into *Escherichia coli* BL21 (DE3) strain for expression. Cells were grown aerobically at 310 K to 3 × 10^8^ cells ml^−1^ followed by induction with 1 m*M* isopropyl-β-d-thiogalactopyranoside and addition of 0.5 m*M* δ-aminolevulinic acid overnight (Sigma-Aldrich, St Louis, USA). Proteins were purified as previously described (Yang *et al.*, 2007 ▸). All steps were performed under green safety light.

### 2.4. Crystallization of SaBphP1-CBD and SaBphP1-PCM constructs   

Crystals of the wild-type SaBphP1-CBD and its T289H mutant proteins were obtained at 289 K in the dark using the hanging-drop vapor-diffusion method at protein concentrations of 10 mg ml^−1^, under the following conditions: 65 m*M* Tris–HCl, pH 8.5, 5.2%(*w*/*v*) PEG 8000, 35%(*v*/*v*) glycerol for the SaBphP1-CBD-wt, and 0.07 *M* sodium acetate trihydrate pH 4.6, 1.4 *M* sodium formate 30%(*w*/*v*) glycerol for the SaBphP1-CBD-T289H. Crystallization of purified SaBphP1-PCM-wt and PCM-T289H was carried out at 291 K using the hanging-drop vapor-diffusion method at a protein concentration of 11 mg ml^−1^ in 0.1 *M* MES (pH 6.2), 8–5%(*w*/*v*) PEG 20 000 and 5.2%(*v*/*v*) acetonitrile. All steps were performed under green safety light. Crystals (about 0.3 mm in all three dimensions, Appendix *B*) were used for X-ray diffraction data collection at 100 K. For the serial femtosecond crystallographic experiments, microcrystals of the SaBphP1-PCM-T289H were prepared by mixing several millilitres of 20 mg ml^−1^ protein with an equal amount of the same precipitant as listed above. The mixture was stirred overnight and left to rest for at least 6 h for the crystals to mature. The microcrystals were concentrated to about 10^11^ crystals ml^−1^ and subsequently folded into a tenfold amount of nuclear grade grease.

### 2.5. X-ray data collection at the Advanced Photon Source and the SACLA XFEL   

X-ray data were collected on macroscopic SaBphP1-wt and T289H mutant CBD and PCM crystals, respectively, at Sector 19, ID-D (Structural Biology Center) of the Advanced Photon Source at cryogenic temperatures (100 K), and processed by *HKL*3000 (Minor *et al.*, 2006 ▸). Data at room temperature were collected from the SaBphP1-PCM-T289H microcrystal–grease mixture at beamline BL3 at SACLA (RIKEN SPring-8 Center, Japan). Up to 400 µl of this mixture were transferred into a reservoir and extruded into air at ambient temperatures (293 K) through a 75 µm wide nozzle with a flow rate of about 5 µl min^−1^. The stream of microcrystals was exposed to intense X-ray pulses of <10 fs duration with a 30 Hz repetition rate. Diffraction patterns were collected on a CCD detector with eight modules (Kameshima *et al.*, 2014 ▸) and analyzed with a user-friendly data-processing pipeline (Nakane *et al.*, 2016 ▸) consisting of hit-finding with *Cheetah* (Barty *et al.*, 2014 ▸), and indexing and Monte Carlo integration by *CrystFEL* (White *et al.*, 2012 ▸). The hit rate was about 15%. Out of 67 175 hits, 36 539 (53%) diffraction patterns were successfully indexed. The extracted partial intensities were merged to full reflection intensities. For data statistics refer to Appendix *B*. The full intensities were converted to structure-factor amplitudes by software based on the *CCP*4 suite of programs (Winn *et al.*, 2011 ▸). Structures were solved as described in Appendix *B*.

### 2.6. Spectroscopy   

We measured UV–vis absorption spectra after irradiation by light-emitting diodes to induce a photoreaction. The first spectrum was collected after the sample was irradiated for 5 min with 740 nm (far-red) light from a fiber-coupled LED (THORLABS M740F2). To enforce the Pr to Pfr photoreaction, the second spectrum was obtained after a 5 min irradiation with 660 nm (red) light using another fiber-coupled LED (THORLABS M660F1). To check for irreversible photo-bleaching, the spectrum was shifted back to Pr for 5 min using the 740 nm LED again (not shown). This protocol resulted in essentially damage-free spectra. If irradiation was extended to >45 min total, substantial chromophore bleaching was observed. The spectra were measured using a Thermo Scientific EVOLUTION 60 spectrophotometer (Fisher, Madison) with a protein concentration of about 0.5 mg ml^−1^. Additional UV–vis absorption spectra on various SaBphP1 constructs were recorded with a different illumination protocol that enhances spectral differences between wild-type and various mutants. Samples were illuminated using light interference filters of 700 and 750 nm, respectively, with a 10 nm bandwidth (ANDOVER, Salem, USA), and the spectra were assayed at room temperature from 240 to 800 nm with a Hitachi 3130 spectrophotometer (Hitachi, Tokyo, Japan). The spectra were normalized, displayed and difference spectra calculated with the free plotting tool *Xmgrace*.

## 3. Results and discussion   

### 3.1. Photomorphogenesis of *S. aurantiaca* and the role of BphPs in fruiting-body formation   

To assess the response of *S. aurantiaca* to light, we irradiated the bacteria under starvation conditions with light of different wavelengths. Orange-colored fruiting bodies were found with blue (450 nm) and far-red (750 nm) light, but not with red light (700 nm) (Fig. 2 ▸ *a*) or in complete darkness (not shown). The red/far-red light response strongly suggests that BphPs, whose structural changes are triggered by these wavelengths, are involved. The *S. aurantiaca* and *C. apiculatus* genome annotations indicate that BphPs are part of a three-gene operon, including a heme oxygenase (BphO), essential in BV synthesis, and a response regulator (RR), apparently forming a typical histidine kinase–RR two-component system (Appendix *A*). The conserved His289 to Thr (or Gly) substitutions are found in BphPs, belonging primarily to myxobacterial species containing multiple BphPs (Appendix *A*). There may be a functional purpose to this substitution, as it has an impact on BphP photoconversion (Mathes *et al.*, 2015 ▸) (see §3.2). BphPs may be acting alone or in concert with other photoreceptors. Besides BphPs, the genomes of *S. aurantiaca* and *C. apiculatus* also contain genes encoding the blue-light photoreceptor known as photoactive yellow protein (PYP) (Fig. 2 ▸ *b*) which may be responsible for the blue-light response described. While the genes coding for BphPs and PYPs mostly appear together and are conserved throughout all three suborders of Myxococcales, they are not present in non-fruiting *Anaeromyxobacter dehalogenans* and the genetically most characterized *M. xanthus* (Fig. 2 ▸ *b*). PYP and BphP signaling pathways are likely to converge as blue and far-red light both stimulate fruiting-body formation (Fig. 2 ▸ *a*). In the purple photosynthetic bacterium *Rhodospirillum centenum* phytochrome-like photoreceptor Ppr contains an N-terminally attached PYP where blue and red signaling pathways combined into a single protein (Jiang *et al.*, 1999 ▸).

### 3.2. Photochemistry of SaBphP1   

**Figure 3:**
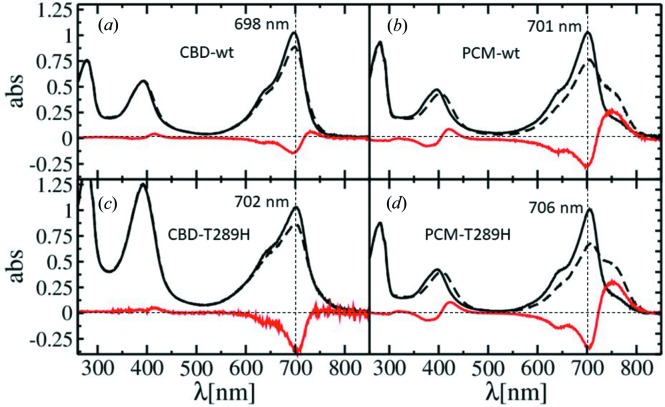
Absorption spectra of SaBphP1 constructs where the Pr to Pfr transition has been initiated with 660 nm light. Black solid line: illumination by 740 nm, black dashed line: illumination by 660 nm, red line: difference spectrum. Absorption maxima wavelengths are marked. Dashed horizontal lines mark zero absorption, vertical dashed lines mark the 700 nm wavelength. (*a*) CBD wild type, (*b*) PCM wild type, (*c*) CBD T289H mutant, (*d*) PCM T289H mutant.

**Figure 4:**
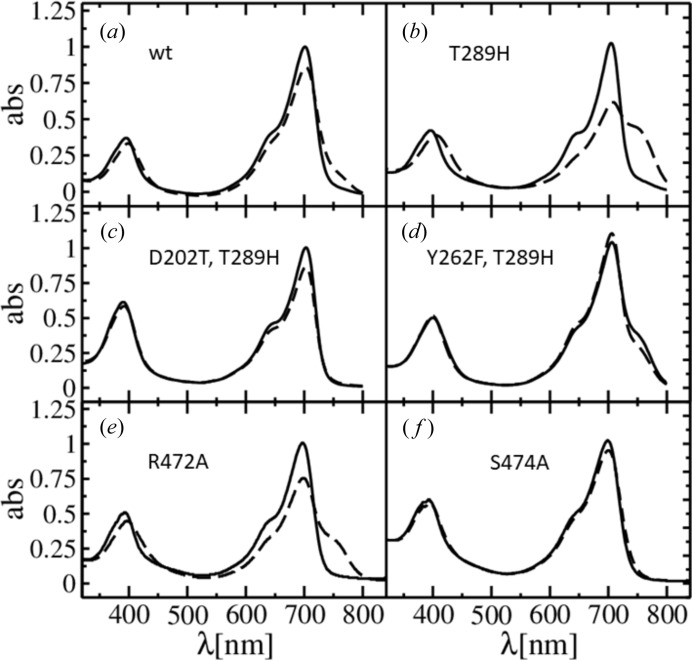
Absorption spectra of various SaBphP1 PCM constructs where the Pr to Pfr transition is initiated with 700 nm light. Black solid line, 20 min of 740 nm; dashed line, 20 min of 700 nm illumination; except (*d*) where illumination is for an hour. The location of the residues can best be identified in, and their function inferred from Figs. 6 ▸ and 9 ▸. (*a*) wild-type, (*b*) T289H mutant, (*c*) D208T, T289H double mutant, (*d*) Y262F, T289H double mutant. Black solid line, dark adapted and 1 h of 700 nm illumination is identical; dashed line, 1 h of 750 nm illumination, (*e*) R472A mutant, (*f*) S747A mutant.

After 660 nm illumination the shorter CBD constructs in *S. aurantiaca* show little photoactivity (Figs. 3 ▸ *a* and 3*c*) as in related BphPs (Wagner *et al.*, 2008 ▸; Yang *et al.*, 2007 ▸). Although bleaching occurs around 700 nm, no characteristic Pfr spectrum is observed. By adding the PHY domain to form the complete PCM module, classical Pfr spectra are obtained after 660 nm light illumination for both the wild-type and the T289H mutant (Figs. 3 ▸ *b* and 3*d*). At this wavelength, the lack of His does not impair photoactivity of the wild-type protein; it slightly alters the spectral shape of the Pfr state (Figs. 3 ▸ *b* and 3*d*). Since the CBDs do not form stable Pfr spectra, but the PCMs do, the PHY domain is essential for the mechanics of the photoswitch. Interestingly, wild-type and mutant PCMs display different spectra following 700 nm illumination so that only a small fraction of the Pfr spectral signature can be identified in the wild-type protein (Figs. 4 ▸ *a* and 4*b*). This different behavior of the wild-type and the mutant is absent following 660 nm light illumination. We provide an explanation for the differences between 660 nm and 700 nm excitation, and the role of the conserved His, based on the SaBphP1 X-ray structures, and comparison with published structures in the Pfr form (see §3.6). Furthermore, an equilibrium between Pr and Pfr states (Figs. 4 ▸ *a* and 4*b*) probably depends on various other factors such as quantum yield, the rate coefficients of the isomerization reaction and the photon fluence, which are outside the scope of this article, and are partially addressed in an earlier publication (Mathes *et al.*, 2015 ▸).

### 3.3. Crystal structures of SaBphP1 wild-type and T289H mutant at cryo and room temperatures   

To gain a detailed understanding of the function of the *S. aurantiaca* phytochromes, we examined the crystal structures of the CBD and the PCM of SaBphP1. We denote these constructs as CBD-wt, CBD-T289H, PCM-wt and PCM-T289H. The wild-type and mutant CBDs both crystallize in the dark in the Pr state in space group *P*3_2_21. Crystals grown from the CBD constructs diffracted to 2.03 Å resolution (Appendix *B*, Table 1 ▸ *b*). The mutant PCM also crystallizes in the dark in the Pr state in *P*3_2_21 with three subunits denoted *A*, *B* and *C* in the asymmetric unit, but the PCM-wt crystallized in space group *C*2 with a single, dimeric BphP molecule in the asymmetric unit. The PCM crystals diffracted to 2.25 and 2.65 Å resolution for wild-type and mutant, respectively (Appendix *B*, Table 1 ▸ *a*). In the mutant PCM crystals, subunits *A* and *B* form a non-crystallographic dimer, and the third subunit, *C*, is related to its mate through a crystallographic twofold symmetry. The density of subunit *C* is not as good as for subunits *A* and *B*, since large parts of the PHY domain are disordered (Appendix *B*, Table 1 ▸ *a*). Using the SACLA XFEL, a data set to 3.15 Å resolution was collected at room temperature on microcrystals of SaBphP1-PCM-T289H (Appendix *B*, Table 1 ▸ *a*).

**Figure 5:**
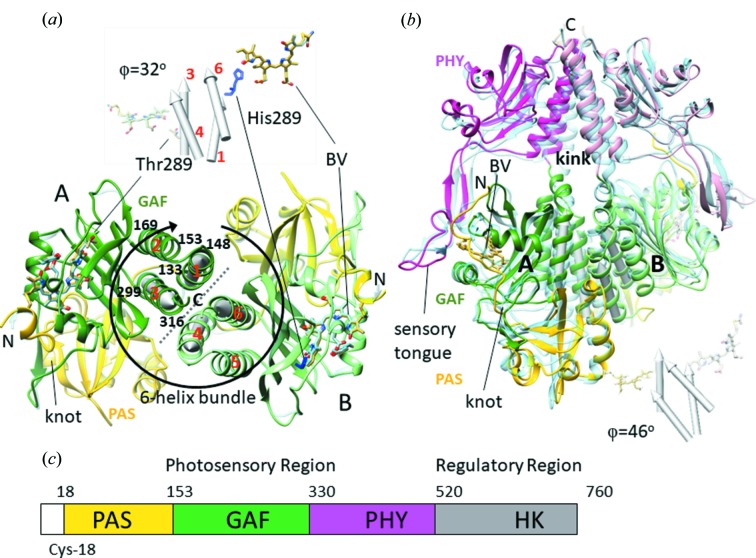
Comparisons of the SaBphP1 CBD (*a*) and PCM (*b*) in the wild-type and T289H mutant forms. The PAS, GAF and PHY domains are colored yellow, green and magenta, respectively. (*a*) Overlay of the wild-type CBD (yellow, green) on the T289H mutant CBD (light blue). The CBD is a dimer (marked A and B) which is generated from the monomer in the asymmetric unit by a crystallographic symmetry operation. Thr289 is marked in subunit A, and His289 in subunit B. The circular arrow denotes the six-helix bundle (helices numbered one to six in red, residue numbers are marked) that forms the dimer interface (gray dotted line). The four interacting helices are highlighted by cylinders, which are also displayed in isolation above the structure. The dimer is rotated so that helices four and six of subunit B lay on top of helices one and three of subunit A. The offset angle φ between the subunits is measured as described in Appendix *B*3. (*b*) Overlay of the wild-type PCM (yellow, green, magenta) on the T289H mutant PCM (light blue). The four helices at the dimer interface are displayed for both constructs below the structure. The angle between the interfaces φ increases by 14° compared with the CBD. The knot, the kink at the helical transition from GAF to PHY and the sensory tongue are marked. (*c*) Domain organization of BphPs. Sequence numbers are provided for SaBphP1.

Structures of the SaBphP1 CBDs and PCMs are shown in Fig. 5 ▸. They display the typical PAS-GAF and PAS-GAF-PHY domain architectures highlighted in yellow, green and magenta, respectively. The BV chromophore is bound to Cys18 close to the N-terminus. The N-terminal amino-acid sequence threads through a loop composed of GAF domain amino acids and forms the knot characteristic of all phytochrome structures. At around residue 320 the GAF domain transitions to the PHY domain. In the wild-type PCM a kinked helical structure is observed, which pivots about residue 325 (Fig. 5 ▸ *b*). The PHY domain harbors the sensory tongue (Fig. 5 ▸ *b*, amino acids 435–485) that is in direct contact with the CBD and reacts to the isomerization of the C15=C16 double bond and rotation of ring *D* transducing the information to the PHY domain (Essen *et al.*, 2008 ▸; Takala *et al.*, 2014 ▸; Burgie *et al.*, 2016 ▸; Anders *et al.*, 2013 ▸; Yang *et al.*, 2008 ▸). Although there is a displacement of the PHY domain caused by the T289H mutant (Fig. 5 ▸ *b*, Appendix *D* and Table 2 ▸), the sensory tongue remains essentially identical in wild-type and mutant SaBphP1.

### 3.4. Structural changes induced by the T289H mutation and the PHY domain   

**Figure 6:**
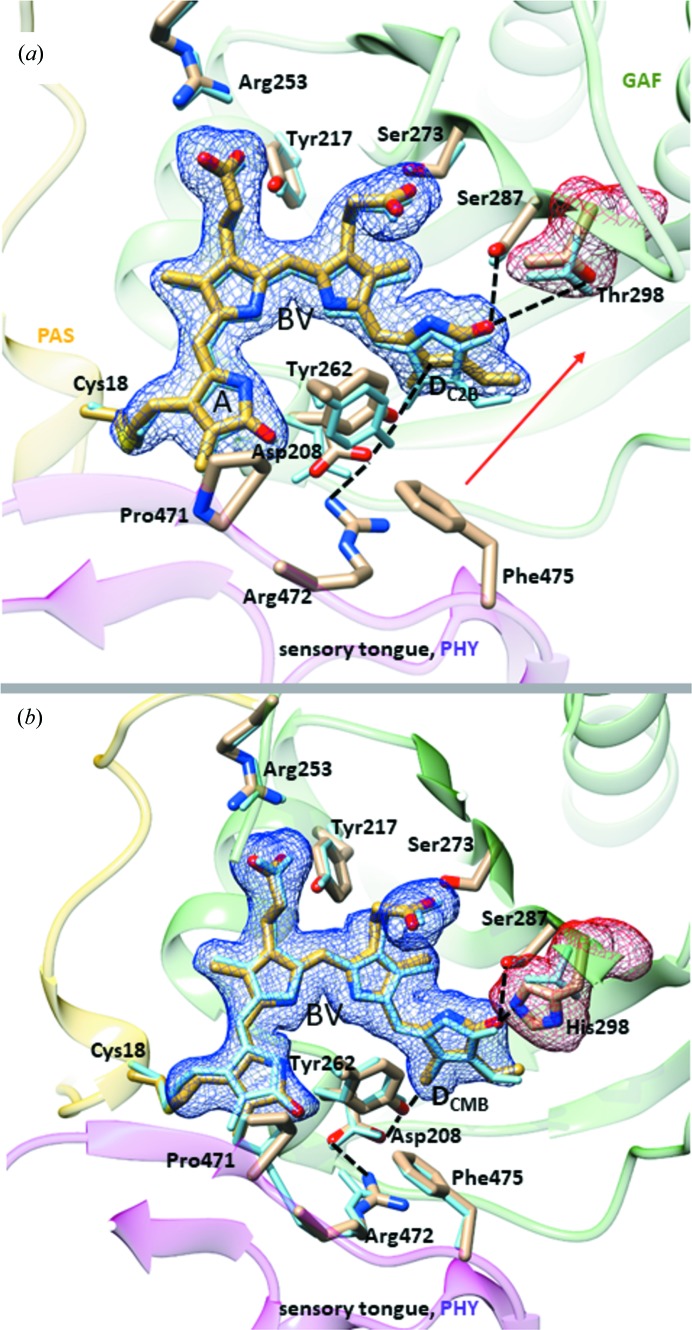
Structural changes in SaBphP1 near the BV chromophore. Important residues are marked. Important salt and hydrogen bonds are shown by dashed lines. (*a*) Effect of adding the PHY domain. Blue atomic structure, CBD-wt; yellow atomic structure, PCM-wt. The electron density (simulated annealing OMIT *mF*_o_ − *DF*_c_, dark blue, contour level: 2σ) is shown for the chromophore and the Thr289 (red). The red arrow indicates the overall trend of the changes. (*b*) Effect of the T289H mutation. Blue atomic structure: PCM-wt, yellow atomic structure: PCM-T289H. The electron density (simulated annealing *mF*_o_ − *DF*_c_, light blue, contour level: 2σ) is shown for the chromophore and the His289 (red).

The structures of the chromophore pocket in SaBphP1 wild-type and T289H mutant CBDs and PCMs are shown in Fig. 6 ▸. When Thr289 is replaced by His, structural displacements throughout the chromophore binding pocket are observed. BV ring *D* moves almost 1 Å deeper into the pocket, and its carbonyl forms a hydrogen bond with His289 (∼2.8 Å) and with Ser287 (3.1 Å). In addition, the electron density of the water that hydrogen bonds to Ser287 near ring *D* is stronger in the mutant and there is an additional water molecule with respect to wild-type suggesting a more rigid and defined BV environment. However, the T289H mutation hardly changes the overall structure of the CBD dimer as the root-mean-square deviation (r.m.s.d.) between wild-type and mutant is on the order of 0.3 Å. The addition of the PHY domain results in a tighter chromophore pocket, while ring *D* is displaced deeper into the GAF domain, and numerous residues such as Asp208 and Tyr262 now make contact with the sensory tongue residue Arg472 of the PHY domain (Fig. 6 ▸ *b*). These structural changes modify the absorption spectra (Fig. 3 ▸), and shift the absorption maxima from 698 nm (for the CBD-wt, Fig. 3 ▸ *a*) to 706 nm (for the PCM-T289H, Fig. 3 ▸ *d*). In addition the orientation of the subunits is changed. For example, the angle φ between the helical bundles at the dimer interface changes from 32 to 46° (see Figs. 5 ▸ *a* and 5*b*, and Table 2 ▸). As a consequence, the CBD dimer cannot be superimposed well on the PCM dimer. Interestingly, the crystallization conditions for the PCM wild-type and T289H mutant are the same, yet they crystallize in different space groups. This already suggests that the mutation affects the relative domain orientation. Indeed, the r.m.s.d. between the T289H mutant and wild-type PCM is ∼3 Å overall. As shown in Fig. 5 ▸(*b*) and Table 2 ▸, the PAS and GAF domains superimpose well, but the PHY domains occupy different positions. The helix near the kink marked in Fig. 5 ▸(*b*) unfolds partially in the mutant, and both PHY domains shift by about 4 Å into the same direction without changing their orientations (Table 2 ▸). Substantial PHY domain structural heterogeneity is observed (as in all BphPs), and is reflected by higher *B* factors compared with the PAS and GAF domains (see Table 1 ▸ *a*).

**Figure 7:**
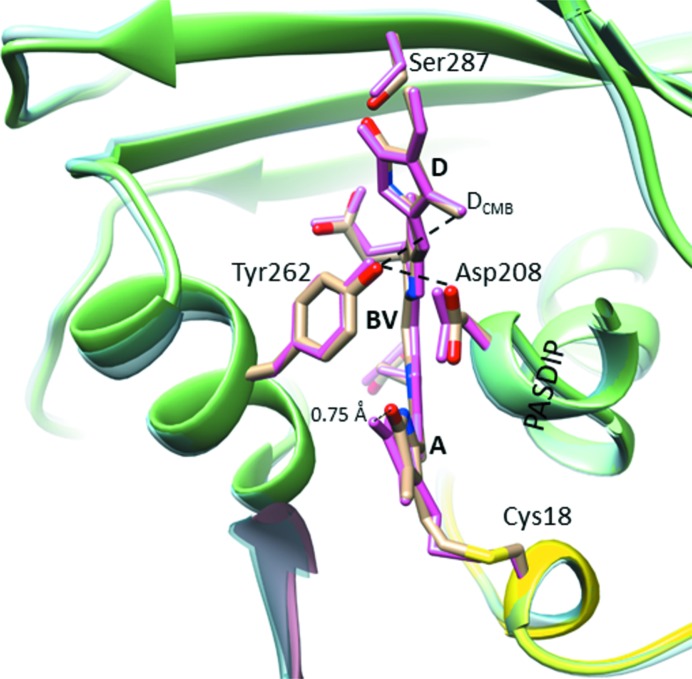
Superposition of the T289H mutant SaBphP1-PCM structures determined at cryo (PAS, yellow; GAF, green; PHY, not shown) and at room temperatures (light-blue ribbon). The chromophore binding site is enlarged and important residues, the PASDIP consensus sequence and the BV chromophore are marked. Dark yellow residues from cryo structure; hot pink from the room-temperature structure.

Cryo-conditions have only a small effect on the overall structure of the SaBphP1-PCM (Fig. 7 ▸). The r.m.s.d. of the T289H-PCMs determined at room temperature and 100 K is only 0.4 Å, not much greater than the coordinate error at 3.1 Å resolution. This demonstrates (i) that freezing does not induce a global structural change, and (ii) that the overall structures of our T289H mutant BphP determined from both microcrystals and macrocrystals are essentially identical. In comparison to the cryo-temperature T289H mutant structure, the BV *A* ring tilts with the covalent bond to the Cys18 S atom displaced at room temperature. However, these local changes might not be significant given the coordinate error at reduced resolution (3.15 Å). Similar local structural differences were observed at substantially better resolution between DrBphP CBD structures determined at cryogenic conditions at the synchrotron (1.35 Å) and at room temperature (2.1 Å) at the Linac Coherent Light Source (LCLS) (Edlund *et al.*, 2016 ▸). In these structures, the cysteine sulfur suffers radiation damage giving rise to structural changes which are also observed here. These small differences, though, might be important for the understanding of the photoswitch, since BV ring *A* is in van der Waals contact with Pro471 which is an important conserved residue in the sensory tongue region of the PHY domain (see below).

It appears as if the PHY domain orientation and details of its tertiary structure are sensitive to the slight structural changes introduced by the T289H mutation near the chromophore (Tables 1*a* ▸ and 2 ▸). PHY displacements between the wild-type and T289H mutant are on the order of 4 Å. This is larger than the mean-square displacement (〈*x* ^2^〉 is about 2 Å) derived from the *B* factor (Table 1 ▸ *a*) and cannot be explained by structural heterogeneity present in the same crystal form. It demonstrates how tightly the PHY domain is coupled to the structural state of the BV chromophore and its immediate environment, a coupling that is essential to a signaling protein. The large 〈*x* ^2^〉 suggests (Table 1 ▸ *a*) that the PHY domains are internally heterogeneous and flexible. Larger structural changes that accompany isomerization from *Z* to *E* as well as the flexibility of the PHY domain are important for complete transition from Pr to Pfr.

### 3.5. The mechanism of the photoswitch revealed by comparison with other X-ray structures   

**Figure 8:**
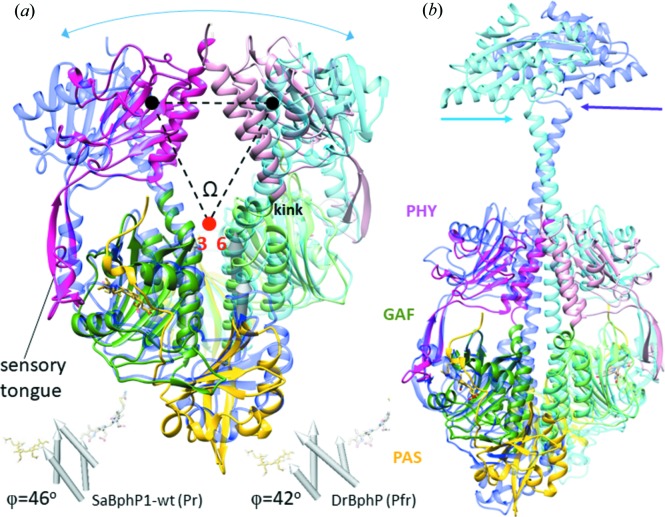
Structural comparison with important BphPs. (*a*) Superposition of the SaBphP1-PCM (Pr) (yellow, green, magenta) onto the DrBphP-PCM (Pfr) in blue (Takala *et al.*, 2014 ▸). The PHY domains are displaced substantially in the Pfr structure (blue curved arrow). The sensory tongue is marked. The four helices at the dimer interface are marked as gray cylinders. The four cylinders are displayed again below the structures (see also Fig. 5 ▸). The offset angle φ between the subunits was measured as described in Appendix *B*3. The approximate positions of the SaBphP-PCM’s PHY domain centroids are shown by black spheres. The opening angle Ω is defined by the centroids and a point between the ends of helices three and six (see also Fig. 5 ▸ *a*). (*b*) Superposition of the SaBphP1-PCM (yellow, green, magenta) on the full length BphP with a di-guanylyl cyclase effector domain from *Idiomarina* sp. (blue) (Gourinchas *et al.*, 2017 ▸).

The PHY domain forms a non-covalently linked extension to the CBD through the sensory tongue (Fig. 5 ▸ *b*) and works in tandem with the GAF domain to tune spectral properties and facilitate physiologically functional photochemistry. In Fig. 8 ▸(*a*), the SaBphP1 PCM crystal structure is compared with the classical *D. radiodurans* BphP in the Pfr state (Burgie *et al.*, 2016 ▸; Takala *et al.*, 2014 ▸). During the Pr to Pfr transition the PHY domains move into opposite directions by 11 Å thereby rotating by 25° (Table 2 ▸) and opening up the dimer interface. The comparison reveals important details of the mechanism of the Pr/Pfr switch. The sensory tongue structure substantially reorganizes between the Pr and Pfr states (Takala *et al.*, 2014 ▸; Burgie *et al.*, 2016 ▸). In the Pr state the tongue region in the PHY domain assumes a loop to β-sheet conformation (Figs. 8 ▸ *a* and 9 ▸ *a*), whereas in the Pfr state, it assumes a loop to α-helix conformation (Figs. 8 ▸ *a* and 9 ▸ *b*). As a consequence, the PHY domains are pulled in opposite directions (see the blue two-sided arrow in Fig. 8 ▸ *a*). The kink between the helices that connect the GAF and PHY domains straightens out. This has a profound influence on the structure and the associated enzymatic activity of the output domain of full length phytochromes (Bjorling *et al.*, 2016 ▸; Burgie *et al.*, 2016 ▸).

**Figure 9:**
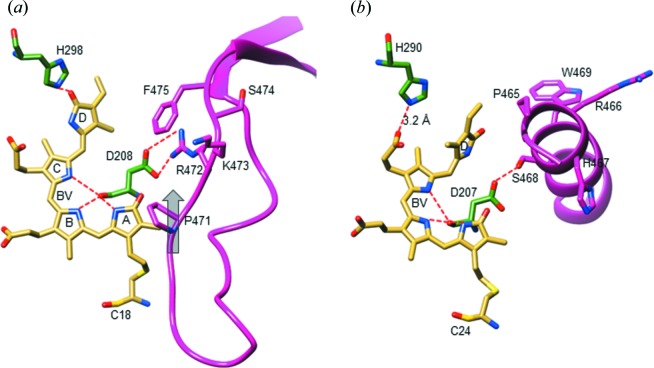
The chromophore and sensory tongue region of the SaBphP1-PCM in the Pr conformation compared with the DrBphP-PCM in the Pfr form. Residues that are considered essential for the Pr to Pfr transition are marked. Red dotted lines: interactions of important residues. (*a*) Chromophore region of the SaBphP1-PCM, BV ring *D* is in the *Z*-configuration. Gray arrow, direction of the structural displacement of P471 from Pr to Pfr. (*b*) Chromophore region of the DrBphP-PCM F469W mutant (Burgie *et al.*, 2016 ▸), ring *D* is in the *E*-configuration. H290 interacts with the ring *D* propionate. Note, compared with the published structure, the His290 ring was rotated by 180° about the χ^2^ angle to motivate the interaction. The DrBphP residues Ser468, Arg466 and Pro465 correspond to Ser474, Arg472 and Pro471 in SaBphP1, respectively (see also Fig. 4 ▸ for absorption spectra of specific mutants).

**Figure 10:**
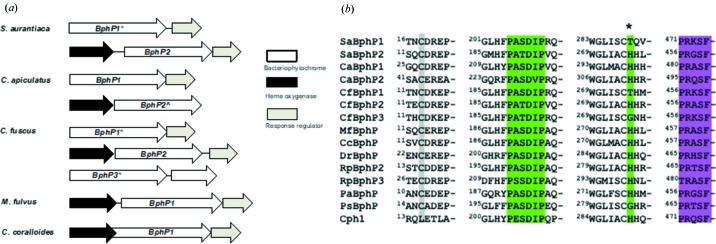
Gene organizations, sequence alignments, consensus sequences, and amino acids relevant for Pr to Pfr transitions. (*a*) Gene organization related to BphPs from *S. aurantiaca* DW4/3–1, *Chondromyces apiculatus*, *Cystobacter fuscus*, *Myxococcus fulvus* HW-1 and *Corallococcus coralloides* DSM 2259 (heme oxygenase opens the tetrapyrrole ring, the response regulator has histidine kinase activity). (*b*) Partial sequence alignment of BphPs. The PASDIP consensus sequence as well as position 289 of the Thr to His mutation in SaBphP1 are marked in green, the PRXSF consensus sequence is marked in pink, the Cys to which the BV is bound is marked in gray. SaBphP1 and SaBphP2 (*S. aurantiaca* DW4/3–1); CaBphP1 and CaBphP2 (*C. apiculatus* DSM 436); CfBphP1, CfBphP2 and CfBphP3 (*C. fuscus*); MfBphP (*M. fulvus* HW-1); CcBphP (*C. coralloides* DSM 2259); DrBphP (*D. radiodurans)*; RpBphP2 and RpBphP3 (*R. palustris* CGA009); PaBphP (*P. aeruginosa* PA01); PsBphP (*Pseudomonas syringae pv. tomato* T1); Cph1 (*Synechocystis* PCC6803).

In SaBphP1 the PHY domain engages the chromophore pocket through a salt bridge between Asp208 and Arg472 (Figs. 6 ▸ *a*, 6*b* and 9 ▸) and seals the BV from contact with the solvent through the sensory tongue. This salt bridge is broken in the Pfr state of the DrBphP as Arg472 (Arg466 in DrBphP) is flipped out to the solvent. Asp208 and Arg472 belong to the highly conserved PASDIP (Wagner *et al.*, 2005 ▸, 2008 ▸; Yang *et al.*, 2007 ▸) and PRXSF (Essen *et al.*, 2008 ▸; Yang *et al.*, 2015 ▸) motifs of the GAF and PHY domains, respectively (Appendix *A*, Fig. 10 ▸ *b*). In addition to Arg472, Asp208 also hydrogen bonds to the conserved Tyr262 (Figs. 6 ▸ *a*, 6*b* and 9 ▸) which is in direct contact with ring *D* and transmits the signal through Asp208 to the sensory tongue. The β-sheet conformation of the sensory tongue is then stabilized through the salt-bridge interaction with Arg472. Additionally, in SaBphP1 Pro471 of the PRXSF motif could also be essential for the sheet-to-helix switch. Pro471 is located in the loop adjacent to the double-stranded β-sheet in the Pr state and at the beginning of the newly formed α-helix in the Pfr state (Fig. 9 ▸ *b*). Proline and glycine are known to destabilize α-helices owing to limited phi/psi space of their dihedral angles. Therefore, when perturbed by the BV ring *D* isomerization, Pro allows for the transition of the β-sheet in the Pr to a helix in the Pfr state. In the unusual RpBphP3 from photosynthetic *Rhodopseudomonas palustris*, which converts from Pr to a near-red absorbing state Pnr (Yang *et al.*, 2015 ▸, 2007 ▸) the amino-acid equivalent to the SaBphP1 Pro471 is actually a threonine (Thr480) (Appendix *A*). To our knowledge, this is the only BphP that lacks a conserved Pro in the PRXSF motif of the PHY domain. In the light of the present results, our observations suggest that the signaling mechanism in RpBphP3 might be distinct from classical BphPs (Yang *et al.*, 2015 ▸). As expected, RpBphP3 becomes a classical Pr/Pfr phytochrome once the Thr in this motif is replaced by Pro (Yang *et al.*, 2015 ▸).

### 3.6. The mechanism of the photoswitch revealed by structural interpretation of absorption spectra obtained from selected SaBphP1 constructs   

Several amino-acid residues including those of the PRXSF motif, play a crucial role during the transition from Pr to Pfr. Conformational changes in the proteins fine-tune spectral forms and promote spectral stability while driven by the isomerization of the bilin from *Z* to *E*. We describe here a sequence of events, initiated by BV ring *D* isomerization and subsequently promoted by several amino acids. This sequence is supported by X-ray structures and corroborated by absorption spectra (Figs. 3 ▸ and 4 ▸) of various SaBphP1 constructs. We show (i) how signaling triggered by ring *D* isomerization can be established, and (ii) how the ring *D* *E*-configuration that causes the formation of the Pfr state can be stabilized.

As mentioned above, the ring *D* trigger can be transmitted *via* Tyr262 and Asp208 towards the sensory tongue residue Arg472 (Figs. 6 ▸ *a* and 6*b*). If Tyr262 is mutated to phenyl­alanine (Fig. 4 ▸ *d*), the configurational space accessible by ring *D* becomes larger, which could change the equilibrium between *Z* and *E* during light illumination. Indeed, the dark-adapted spectrum of the Y262F T289H double mutant (with His289 present to stabilize ring *D*) is essentially identical to the one obtained after illumination with 700 nm (Fig. 4 ▸ *d*). This effect is even more pronounced in the Y262F single mutant (spectrum not shown) where ring *D* is less restrained, and could access an even larger configurational space. We presume that the configuration of ring *D* cannot be sensed in this mutant, and the spectrum obtained (Fig. 4 ▸ *d*) is probably caused by a benign thermal isomerization of ring *D* without concomitant structural transitions. The mutation of Asp208 to threonine (D208T) abolishes the formation of a stable *E*-configuration altogether (Fig. 4 ▸ *c*). A perturbation would quickly revert back to the *Z*-configuration. Accordingly, Asp208 effectively promotes the structural transition from Pr to Pfr.

As shown by published X-ray structures, the β-sheet to α-helix transition of the tongue leads to a stabilization of the chromophore *E*-isomer and its associated spectrum, resulting in a large displace­ment of the PHY domain in the Pfr state (Takala *et al.*, 2014 ▸; Burgie *et al.*, 2016 ▸). In the Pfr structures of BphP from *P. aeroginosa* and *D. radiodurans* (Yang *et al.*, 2008 ▸; Burgie *et al.*, 2016 ▸; Takala *et al.*, 2014 ▸), the His, which is equivalent to His289 in our structures, interacts with the ring *C* propionate (Fig. 9 ▸ *b*). When this His is absent, as in the SaBphP1-PCM-wt, the formation and stabilization of the Pfr state is impeded. As a result, the sensitivity to light excitation is different for the SaBphP1 mutant and the wild-type (Figs. 3*b*, 3*d* ▸, 4*a* and 4*b* ▸). Ser474 which is also part of the PRXSF motif is located in the helical segment of the sensory tongue in the Pfr state of DrBphP. It forms a hydrogen bond with ring *D* in the *E*-configuration (Takala *et al.*, 2014 ▸; Burgie *et al.*, 2016 ▸) (Fig. 9 ▸ *b*). In the SaBphP1 Ser474Ala mutant, the weak Pfr state spectral signature that can be seen in the wild type is absent (compare Figs. 4 ▸ *a* and 4*f*). We speculate that the sensory tongue helix is transiently forming, but ring *D* in the *E*-configuration cannot be stabilized, which would prevent the development, accumulation and stabilization of the Pfr conformation. Interestingly, if residue Arg472 (Figs. 6 ▸ *a*, 6*b* and 9 ▸) is mutated to alanine (the R472A mutant in the Thr298 wild-type background), a stable Pfr formation is restored at 700 nm excitation (Fig. 4 ▸ *e*). We suspect that the nearby sensory tongue residue Lys473 (Figs. 8 ▸ and 9 ▸ *c*) may flip into place in the R472A mutant and functionally replace both Arg472 and His289 by promoting a stable chromophore *E*-configuration. Further confirmation of our hypothesis awaits the R472A mutant structure determination. In Pfr, Arg472 points away from the chromophore pocket (see the DrBphP Arg466 in Fig. 9 ▸ *b*) and it is not involved in the stabilization of the ring-*D* *E*-configuration and the concomitant formation of Pfr.

In summary, from a comparison of SaBphP1 and published BphP structures, and interpretation of absorption spectra of specific mutants, we have identified a network of conserved amino-acid residues in the myxobacterial BphP1 that sense BV isomerization, trigger the β-sheet to α-helix transition of the sensory tongue, and stabilize Pr and Pfr conformations associated with the *Z* or *E* chromophore isomeric forms.

## 4. Outlook   

An important goal must be to determine a structure of the full length SaBphP1 (wild type and/or T289H mutant) including its HK output domain in both the Pr and Pfr forms at high resolution. The phosphorylation states of the HK domain in both SaBphP1 wild type and in mutant Pr and Pfr must be determined *in vivo*. Perhaps most importantly, the structural and biophysical results must be linked to bacterial photomorphogenic behavior. For example, the phytochrome genes need to be selectively eliminated from the bacterial genome, and the alterations, if any, in the light-dependent physiological response of *S. aurantiaca* and/or related *M. fulvus* that contains only one BphP and no PYPs, must be demonstrated. A more ambitious goal is to observe the photo-half-cycles of the phytochromes by time-resolved crystallographic methods and characterize the transmission of signal from the BV to the enzymatic domain through a distance of about 150 Å of protein (Fig. 8 ▸ *b*), in real time. Recent results obtained by time-resolved serial femtosecond crystallography (TR-SFX) (Aquila *et al.*, 2012 ▸) at the LCLS (Tenboer *et al.*, 2014 ▸; Barends *et al.*, 2015 ▸; Pande *et al.*, 2016 ▸) and at SACLA (Nango *et al.*, 2016 ▸) suggest that these types of experiments are also feasible with SaBphP1. The T289H mutant microcrystals diffracted beyond 3 Å at SACLA in air, and resolution should improve when the samples are (i) injected into a vacuum where the background is greatly reduced, and (ii) Bragg intensities can be measured to higher resolution. The resolution achieved with wild-type SaBphP1-PCM (2.25 Å) is the highest so far reached for unmodified PCM dimers. Hence, the SaBphP1-PCM crystals are perfectly suited to reach the near atomic resolution range (∼2.5 Å) in TR-SFX experiments. At this resolution, difference electron-density maps will contain chemically meaningful features (Tenboer *et al.*, 2014 ▸) and can be used to identify and characterize the structures of transient intermediates during the Pr to Pfr transition.

Since at least some of the conformational changes between the Pr and Pfr states in these light regulated enzymes are large (several tens of Å; Burgie *et al.*, 2016 ▸; Gourinchas *et al.*, 2017 ▸; Bjorling *et al.*, 2016 ▸), it has to be determined whether crystalline and/or microcrystalline lattices are compatible with these structural changes. X-ray technology is advanced enough that structures can be determined from solvated single virus particles which are injected into the ultra-intense X-ray pulse of an XFEL (Munke *et al.*, 2016 ▸; Hosseinizadeh *et al.*, 2017 ▸). Therefore, we anticipate that single-particle X-ray experiments on the intact (full-length) phytochromes such as that shown in Fig. 8 ▸(*b*) can be conducted at moderate atomic resolution at tender X-ray sources such as the upcoming LCLS-II (Heimann *et al.*, 2018 ▸). Thus, we expect that the observation of the unconstrained, fast and ultrafast structural dynamics of the Pr to Pfr transition in this important class of light-regulated enzymes may become a reality. 

## Supplementary Material

PDB reference: chromophore binding domain of *Stigmatella aurantiaca* phytochrome P1 wild-type, 6baf (SaBphP1-CBD-wt)

PDB reference: chromophore binding domain of *Stigmatella aurantiaca* phytochrome P1 T289H mutant, 6bak (SaBphP1-CBD-T289H)

PDB reference: wild-type photosensory core module for *Stigmatella aurantiaca* phytochrome P1, 6bao (SaBphP1-PCM-wt)

PDB reference: photosensory core module for *Stigmatella aurantiaca* phytochrome P1 T289H mutant, 6bap (SaBphP1-PCM-T289H)

PDB reference: photosensory core module for *Stigmatella aurantiaca* phytochrome P1 T289H mutant at room temperature, 6bay (SaBphP1-PCM-T289H)

## Figures and Tables

**Figure 11:**
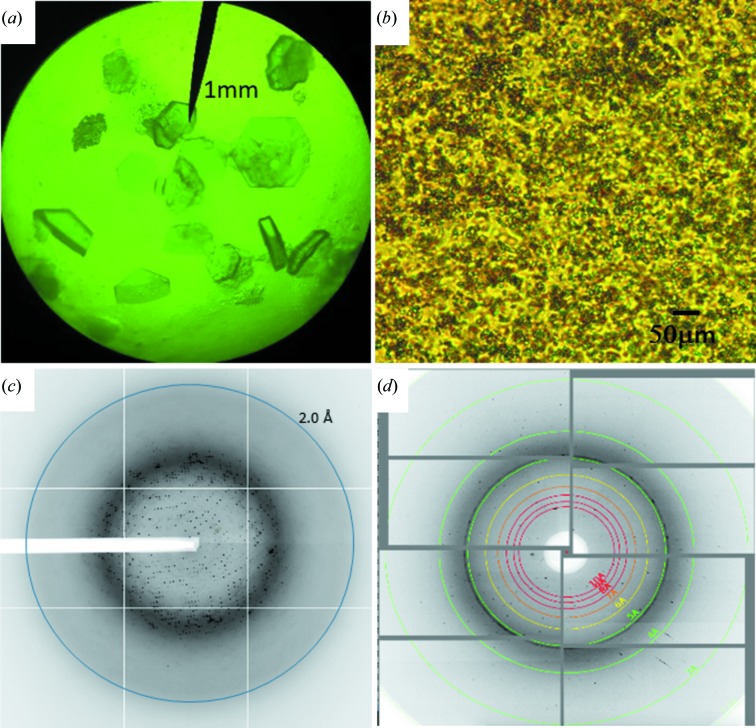
Examples of SaBphP1 crystals and diffraction patterns. (*a*) Wild-type PCM crystals, (*b*) T289H mutant PCM microcrystals as used at SACLA, (*c*) wild-type PCM diffraction pattern obtained at the synchrotron (APS, 19-ID-D), (*d*) T289H mutant diffraction pattern from SACLA.

**Figure 12:**
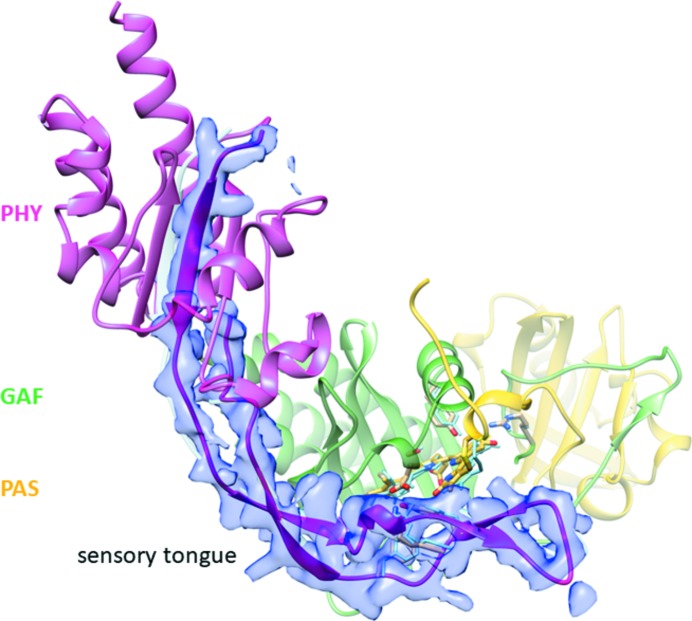
The sensory tongue in the SaBphP1-PCM wild-type and Thr289His mutant. The region from residue 435 to 490 in the PCM of the T289H mutant, subunit *B*, is displayed in transparent blue (2*mF*_o_ − *DF*_c_ electron density at the 1.1σ contour level). The PAS, GAF and PHY domains are colored yellow, green and magenta, respectively.

**Table:** The data in the highest resolution shell are given in parentheses. Resolution limit is estimated from CC_1/2_ > 1/e (36.8%). The *D*_CC = 0.5_ indicates that there is information in the data beyond our chosen resolution limit.(*a*) Structures of the SaBphP1 PCM wild-type and T289H mutant at cryogenic and room temperatures.

	SaBphP1- 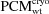		SaBphP1- 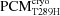			SaBphP1- 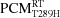		
Beamline	APS 19-ID-D		APS 19-ID-D			SACLA-BL3		
Resolution (Å)	44.2–2.25		35.2–2.65			53.8–3.15		
Temperature (K)	100		100			293		
Space group	*C*2		*P*3_2_21			*P*3_2_21		
Unit-cell parameters (Å,°)	*a* = 82.5, *b* = 135.1, *c* = 113.2, α = 90, β = 105.9, γ = 90		*a* = 83.5, *b* = 83.5, *c* = 475.4, α = 90, β = 90, γ = 120			*a* = 84.2 *b* = 84.2, *c* = 478.0, α = 90, β = 90, γ = 120		
No. of reflections observed/unique	267825/55416		200661/56754			21656134/41044		
Redundancy	4.8 (4.1)		3.5 (3.3)			527 (197)		
Completeness (%)	97.4 (96.8)		99.1 (99.2)			100		
CC_1/2_ (%)	99.8 (39.9)		99.7 (38.0)			99.6 (37.0)		
〈*I*/σ(*I*)〉	8.3 (0.8)		7.0 (0.8)			4.8 (0.8)		
*R*_split_ (%)	—		—			9.3 (176.0)		
*R*_merge_ (%)	5.8 (100.5)		6.8 (55.9)			—		
Refinement, *PHENIX* †	—		—			—		
*R*_cryst_, *R*_free_	21.9 (36.1), 26.5 (38.4)		24.6 (29.8), 31.5 (38.8)			21.6 (31.1), 27.8 (42.1)		
*D*_CC = 0.5_ (Å)‡	2.15		2.59			—		
No. of residues/subunit, No. of subunits/asymmetric unit	504, 2		504, 3			504, 3		
No. of water molecules	118		51			—		
R.m.s. deviations: bonds (Å), angles (°)	0.010, 1.24		0.013, 1.57			0.010, 1.37		
〈*B* factor〉 (Å^2^)	*A*	*B*	*A*	*B*	*C*	*A*	*B*	*C*
PAS-GAF	54	57	67	66	95	114	110	146
PHY	100	82	97	90	174	124	142	207

**Table:** (*b*) Structures of the SaBphP1 CBD wild-type and mutant.

	SaBphP1- 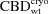	SaBphP1- 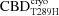
Beamline	APS 19-ID-D	APS 19-ID-D
Resolution (Å)	23.9–2.25	24.0–2.03
Temperature (K)	100	100
Space group	*P*3_2_21	*P*3_2_21
Unit-cell parameters (Å,°)	*a* = 131.7, *b* = 131.7, *c* = 96.1, α = 90, β = 90, γ = 120	*a* = 131.4, *b* = 131.4, *c* = 95.7, α = 90, β = 90, γ = 120
No. of reflections observed/unique	197217/45439	255135/60666
Redundancy	4.3 (4.3)	4.2 (4.3)
Completeness (%)	98.4 (98.4)	98.7 (98.4)
CC_1/2_ (%)	99.5 (38.2)	99.7 (41.6)
〈*I*/σ(*I*)〉	6.3 (0.8)	6.4 (1.0)
*R*_merge_ (%)	7.8 (55.7)	7.8 (89.3)
Refinement, *PHENIX* †	—	—
*R*_cryst_, *R*_free_	21.1 (31.1), 22.8 (34.0)	20.6 (32.9), 22.5 (34.1)
*D*_CC = 0.5_ (Å) ‡	2.07	1.90
No. of residues/subunit, No. of subunits/asymmetric unit	302, 1	302, 1
No. of water molecules	214	321
R.m.s. deviations: bonds (Å), angles (°)	0.023, 2.66	0.019, 1.89
〈*B* factor〉 (Å^2^)	50.5	48.6

† The highest resolution of the refinement set to the highest resolution of the data (given by CC_1/2_).

‡ The resolution at which the correlation coefficient between observed and calculated amplitudes reaches 0.5.

**Table 3:** Absorption maxima (in nm) for wild-type and T289H mutant of SaBphP1

SaBphP1	Wild-type	T289H mutant
CBD (PAS-GAF)	698	702
PCM (PAS-GAF-PHY)	701.5	706

**Table 2:** Geometric analysis of selected BphP structures as compared with the SaBphP1-PCM-wt Resolution limits *d*_min_ are indicated. The superposition was performed by *Chimera* (Pettersen *et al.*, 2004 ▸) using Smith–Waterman alignment of C^α^ atoms (outliers >5 Å were ignored). IF, angle of relative subunit orientations at the interface; *d*-centroids, distance between PHY domain centroids, the opening angle Ω is estimated as explained in the text and in Fig. 8 ▸(*a*). The PHY domain rotation was calculated as explained. Last column: similarities (root-mean-square displacement) of PAS-GAF domain C^α^ atomic positions relative to SaBphP1-PCM-wt as reference. AP, antiparallel orientation; nd, not done; ld, incomplete PHY domain.

Organism, specifics	PDB entry	*d*_min_ (Å)	IF (°)	*d*-centroids, Ω (Å,°)	PHY rotation (°)	R.m.s.d. (Å)
*S. aurantiaca,* wt	This study	2.25	46	30, 54	ref.	ref.
*S. aurantiaca,* T289H	This study	2.65	46	26, 44	2	0.9
*Agrobacterium tumefaciens* †	5hsq	1.85	AP	nd	23	1.4
*Deinococcus radiodurans,* Pr	4q0j	2.75	45	35, 66	8	1.4
*D. radiodurans,* Pfr	4o01	3.24	42	46, 85	33	1.8
*P. aeruginosa* ‡	3nhq	2.55	10	25, 4	23	1.2
*Idiomarina* sp.§	5llw	3.00	37	27, 54	30	1.6
*Synechocystis*	2vea	2.21	AP	nd	14	1.3
*Arabidopsis*	4our	3.4	28	ld	45	1.4

† Surface mutations to support crystallization with anti-parallel subunit orientation.

‡ Unusual phytochrome, dark-adapted Pfr form.

§ Full length, ∼620 amino acids.

## Appendix A Protein sequence alignment

The organization of genes related to BphPs in the *Cystobacterineae* suborder of the Myxococcales is depicted in Fig. 10 ▸(*a*). Partial protein-sequence alignments are displayed in Fig. 10 ▸(*b*), and were generated with the *Basic Local Alignment Search Tool* (*BLAST*) and/or *Clustal Omega*. The PASDIP and the PRXSF consensus sequences are conserved through all bacterial phytochromes (Fig. 10 ▸ *b*).

## Appendix B

### b1. Crystal forms and diffraction patterns

Macroscopic crystals of SaBphP1-PCM(wt) as used to collect data at APS, ID-19 are shown in Fig. 11 ▸(*a*). Microcrystals of the T289H mutant PCM (Fig. 11 ▸ *b*) are injected into the SACLA beam at experimental station BL3. Their respective diffraction patterns are depicted in Figs. 11 ▸(*c*) and 11(*d*). Note that the MPCCD detector at SACLA station BL3 was able to resolve the long axis (475 Å) of the SaBphP1-PCM-T289H microcrystals. The outer ring (in faint green) corresponds to 3 Å resolution.

### b2. Structure determination and refinement of the SaBphP1 CBD constructs

The crystal structure of the wild-type SaBphP1-CBD was determined by the molecular replacement method using *MOLREP* (Vagin & Teplyakov, 2010 ▸) implemented in *HKL*3000 and the crystal structure of the RpBphP3-CBD (PDB code 2ool; Yang *et al.*, 2007 ▸) as a search model. The crystal structure of the SaBphP1-CBD-T289H was determined by using the crystal structure of SaBphP1-CBD-wt as a search model. Both initial models of SaBphP1 were checked and corrected in *Coot* (Emsley *et al.*, 2010 ▸). The crystal structures were initially refined with *REFMAC* v.5.5 (Murshudov *et al.*, 2011 ▸) from the *CCP*4 package (Winn *et al.*, 2011 ▸). Water molecules were added, and the final structure refined using *PHENIX* (Adams *et al.*, 2010 ▸). The quality of the final models was checked with the validation tool in *PHENIX* based on *MolProbity* (Chen *et al.*, 2010 ▸). Data collection and refinement statistics are given in Table 1 ▸(*b*).

### b3. Structure determination of the SaBphP1 PCM constructs

To determine the wild-type PCM structure, the SaBphP1-CBD-wt monomer was used for initial molecular replacement using *PHASER* (Oeffner *et al.*, 2013 ▸). A solution consisting of a dimer (PAS-GAF_*A*,*B*_) was found. The PHY domain was extracted from the *D. radiodurans* PCM (PDB code 4q0j; Burgie, Wang *et al.*, 2014 ▸) starting from residue 325. Two straightforward solutions were found (*R*_cryst_ = 37%) comprising a PAS-GAF-PHY dimer in the asymmetric unit of the monoclinic crystals. The structure was refined with the programs *REFMAC*5 (Murshudov *et al.*, 2011 ▸) and *PHENIX* (Adams *et al.*, 2010 ▸) initially with rigid-body refinement defining each of the PAS (residues 17–136), GAF (residues 137–320) and PHY (residues 324–502) domains as rigid entities. Additional electron density that appeared in the chromophore binding pocket was interpreted by a biliverdin linked to Cys18. All gaps in the search model could be closed by tracing the electron density with the gap-filling tool implemented in the program *Coot* (Emsley *et al.*, 2010 ▸). After the refinement converged, solvent molecules were added using *Coot*. In addition, electron density appeared at the N-terminal end of the structure which was interpreted as amino acids 11–16. The SaBphP1 wild-type PCM structure () consists of 511 amino acids that trace the PAS, GAF and PHY domains without gaps, the biliverdin and water molecules. To find a molecular replacement solution for the SaBphP1-PCM-T289H mutant data, the wild-type PCM subunit *A* was used as a search model. The PHY domain was removed (residues 324–511). Three solutions (PAS-GAF_*A*,*B*,*C*_) were found in the asymmetric unit. A molecular replacement solution for the PHY domain fragment (residues 326–511) was determined with PAS-GAF_*A*,*B*,*C*_ kept fixed. PHY domain solutions were found for PAS-GAF_*A*_ and PAS-GAF_*B*_ (*R*_cryst_ = 44%). Although the electron density of the PAS and GAF domains in subunit *C* was distinct, the electron density of the PHY domain was weak and almost non-existent except at the sensory tongue. To construct a comprehensive model for the *C* subunit, the complete solution for subunit *A*, which includes the PHY domain, was least-squares fitted using common residues onto the molecular replacement solution PAS-GAF_C_. The resulting structure was refined using *PHENIX* (Adams *et al.*, 2010 ▸). Subunits *A* and *B* form a dimer in the asymmetric unit, subunit *C* contributes to a disordered dimer through a crystallographic symmetry operation. Difference electron density at position 289 identifies the mutation. Threonine was replaced by histidine using *Coot*. All loops were inspected and their structures corrected manually if necessary, since different crystal contacts in the mutant *P*3_2_2 crystals and the wild-type make additional structural differences likely. To determine the room-temperature structure of the SaBphP1-T289H-PCM (), the  was used as an initial model. This model was refineable against the SACLA data resulting in acceptable *R*_cryst_ values and no apparent and strong difference electron-density features. Table 1 ▸(*a*) shows data collection and refinement statistics. The simulated-annealing OMIT maps in Fig. 6 ▸ were calculated in *PHENIX* by omitting the chromophore and (His/Thr)289. The offset angle φ between the subunits is measured using the ‘axis’ option in the program *Chimera* (Pettersen *et al.*, 2004 ▸), and averaging the angles between the dimer interface helices (Figs 5 ▸ and 8 ▸ *a*). Fig. 12 ▸ shows exemplary 2*mF*_o_ − *DF*_c_ electron density near the sensory tongue of the PCM-T289H mutant.

## Appendix C Absorption maxima of the SaBphP1 wild-type and T289H mutant constructs

The absorption maxima shifts between the CBD and PCM of the wt and T289H mutant forms in Table 3 ▸ (see also Fig. 3 ▸) can be qualitatively understood by slight changes in the chromophore geometry as well as the extension of a mesomerically coupled π-system by hydrogen bonds as suggested in Schmidt *et al.* (2007 ▸). The His289 mutation extends the effective size of the chromophore π-system, and the addition of the PHY domain straightens out the BV chromophore compared with the wild-type. Both effects lead to shifts to higher wavelengths. Accordingly, if compared with the other three constructs, the spectrum of the SaBphP1-PCM-T289H is found at larger wavelengths.

## Appendix D Comparison of phytochrome structures determined here with selected structures from the literature

The Protein Data Bank (Berman *et al.*, 2002 ▸) contains ∼50 phytochrome structures. Most are CBDs in the Pr state, of which *D. radiodurans* BphP (DrBphP) structure was the first example (Wagner *et al.*, 2005 ▸). BphP PCM structures were solved more recently from *Agrobacterium fabrum C58* (formerly *A. tumefaciens C58*) (Nagano *et al.*, 2016 ▸), *R. palustris* (Yang *et al.*, 2015 ▸) and from cyanobacteria (Essen *et al.*, 2008 ▸), all in the dark-adapted Pr state. A full-length BphP (in its dark-adapted Pr state) is available from *Xanthomonas campestris* (Otero *et al.*, 2016 ▸), and most recently with known enzymatic activity, di-guanylyl cyclase, from *Idiomarina* (Gourinchas *et al.*, 2017 ▸) (Fig. 8 ▸ *b*). DrBphP PCM structures are known in both the dark-adapted Pr and the light-activated Pfr states (Takala *et al.*, 2014 ▸; Burgie *et al.*, 2016 ▸). Unlike other BphPs, the dark-adapted *P. aeruginosa* bathyphytochrome is in the *E*-configuration (Pfr state) (Yang *et al.*, 2011 ▸). The transition of the PCM of this phytochrome from Pfr to its light activated *Z-*configuration (Pr) could be partially identified by cryo-trapping kinetic crystallography (Yang *et al.*, 2011 ▸). Pairwise structural alignments were performed using Smith–Waterman alignment of C^α^ atoms implemented into the program *Chimera* (Pettersen *et al.*, 2004 ▸). Results are shown in Table 2 ▸. If dimers were compared, differences in the interface and the PHY domain orientations would result in r.m.s.d. values larger than 5 Å. Instead, single PCM subunits consisting of PAS-GAF-PHY domains were oriented onto each other by fitting PAS-GAF regions only. R.m.s.d.s were excellent through all phytochromes (see the last column of Table 2 ▸). After this, differences in the orientation of the PHY domains were evaluated separately by comparing the PHY domain of the fitted PCM subunit to the one of the reference. For convenience, a Python script that employed an approach based on the singular value decomposition to generate the rotation matrix that relates the subunits was used. Results were identical to the output of the program *lsqman* (Kleywegt & Jones, 1995 ▸). Geometric centers (centroids) were calculated for the two PHY domains, each, in the dimers using also a python script. The sensory tongue was omitted from the calculation. Short centroid distances indicate closely spaced PHY domains, whereas large centroid distances indicate an open, Pfr-like configuration. The opening angle was estimated from the centroid positions and a point that is approximately equivalent to a point at the end of the dimer interface as indicated in Fig. 8 ▸(*a*). The structural figures were generated using *Chimera* (Pettersen *et al.*, 2004 ▸) and *PyMOL* (DeLano, 2002 ▸). Although the phytochromes are the most important red-light photoreceptors in plants, they have proved much more difficult to crystallize than BphPs and only one structure is known, that of the PCM from *Arabidopsis thaliana* phytochrome B (PhyB) PCM (Burgie, Bussell *et al.*, 2014 ▸; Legris *et al.*, 2016 ▸). The SaBphP1-PCM-wt can be best overlaid with the DrBphP in the Pr form which features a similar centroid distance, and a difference of only 8° of PHY domain rotation (Table 2 ▸). In the Pfr form the PHY domains are largely displaced resulting in a Y-shaped overall structure (Takala *et al.*, 2014 ▸; Burgie *et al.*, 2016 ▸) with reoriented PHY domains (33° difference to the SaBphP1-wt PHY domain), and an opening angle of 85°.
